# Network Toxicology and Molecular Docking Reveal the Toxicological Mechanisms of DEHP in Bone Diseases

**DOI:** 10.3390/ijms262210895

**Published:** 2025-11-10

**Authors:** Zhonghao Fan, Haitao Du, Xinyi Zhou, Cheng Wang, Mengru Zhang, Tiefeng Sun, Yi Wang, Ping Wang

**Affiliations:** 1 School of Pharmacy, Shandong University of Traditional Chinese Medicine, Jinan 250355, China; fzh01225@163.com (Z.F.); 15753332285@163.com (X.Z.); 2 Institute of Traditional Chinese Medicine Pharmacology (Pharmacological Experimentation Center), Shandong Academy of Chinese Medicine, Jinan 250014, China; kkitdht@foxmail.com (H.D.); 18306390275@163.com (C.W.); 15625156296@163.com (M.Z.); suntiefenglove@163.com (T.S.)

**Keywords:** Di(2-ethylhexyl) phthalate (DEHP), network toxicology, molecular docking, osteoporosis, osteoarthritis, osteonecrosis of the femoral head

## Abstract

Di(2-ethylhexyl) phthalate (DEHP), a widely employed exogenous plasticizer, has become pervasive in the environment and living organisms due to its extensive use in food packaging, medical devices, and daily consumer products, and is established as a typical endocrine-disrupting chemical. Growing evidence indicates a strong association between DEHP exposure and the incidence of chronic bone disorders, including osteoporosis (OP), osteoarthritis (OA), and osteonecrosis of the femoral head (ONFH). However, the molecular mechanisms underlying its pathogenic effects across these diseases remain poorly defined. In this study, we applied an environmental network toxicology approach to integrate predicted protein targets of DEHP with known disease-associated targets of the three bone disorders using multiple databases. Through Venn analysis, protein–protein interaction (PPI) network construction, and Gene Ontology (GO) and Kyoto Encyclopedia of Genes and Genomes (KEGG) enrichment analyses, we identified core targets and key signaling pathways. Molecular docking and molecular dynamics (MD) simulations were further employed to validate the binding modes and stability between DEHP and the core targets, thereby elucidating common and distinct mechanisms of DEHP across these bone diseases. A total of 109 overlapping targets of DEHP and the three bone diseases were identified, among which 7 core targets—AKT1, SRC, ESR1, CASP3, MMP9, BCL2, and BCL2L1—were common to all three disorders. These are implicated in critical biological processes such as apoptosis regulation, inflammation, extracellular matrix degradation, and estrogen signaling. KEGG enrichment analysis revealed significant involvement of the PI3K-Akt, MAPK, Ras, TNF, and estrogen signaling pathways across all three diseases. Molecular docking and MD simulations confirmed stable binding of DEHP to key targets including AKT1, ESR1, and MMP9, supporting its potential to disrupt bone metabolic homeostasis via multi-target and multi-pathway mechanisms. Further analysis indicated that DEHP exerts both shared and disease-specific effects: it disrupts osteoblast/osteoclast balance in OP, amplifies inflammatory responses and matrix degradation in OA, and contributes to impaired angiogenesis and osteocyte necrosis in ONFH. This study systematically reveals how DEHP disrupts bone homeostasis through a multi-target and multi-pathway network, constructing a cross-disease osteotoxicity framework. It is the first to delineate the common and distinct molecular mechanisms of DEHP in OP, OA, and ONFH. Although these insights are derived from computational models and require further experimental validation, they provide a novel theoretical basis for combined intervention strategies targeting multiple bone diseases and for environmental health risk assessment.

## 1. Introduction

Di(2-ethylhexyl) phthalate (DEHP) is the most widely used and highest-produced phthalate compound. As a typical exogenous plasticizer, DEHP is primarily added to polyvinyl chloride (PVC) and various other polymer materials to significantly enhance their flexibility and plasticity. However, numerous studies have demonstrated that DEHP exhibits significant endocrine-disrupting effects and is therefore classified as an important endocrine-disrupting chemical (EDC) [1]. Structurally, DEHP contains a phthalic acid backbone and two branched 2-ethylhexyl groups, which are not chemically bonded but physically mixed into polymers, leading to its tendency to leach out during use and storage [2]. This property facilitates its widespread presence in food contact materials, single-use medical devices (e.g., infusion tubes and blood bags), children’s toys, and cosmetics [3]. As a result, DEHP is ubiquitous in daily living environments, where it persists with high stability and resistance to degradation, contributing to environmental residue and bioaccumulation [4,5]. Upon entering the body, DEHP is readily hydrolyzed by nonspecific esterases into mono(2-ethylhexyl) phthalate (MEHP), which exhibits higher bioactivity and mediates diverse toxic responses through multiple signaling pathways [6]. In the reproductive system, DEHP/MEHP can inhibit key steroidogenic enzymes (e.g., StAR, CYP11A1, and CYP17A1) and interfere with androgen and estrogen receptor pathways, thereby impairing reproductive endocrine function and causing issues such as abnormal spermatogenesis, testicular atrophy, and ovarian dysfunction [7,8,9]. In the metabolic system, DEHP/MEHP primarily acts on peroxisome proliferator-activated receptors (PPARα/γ), disrupting lipid metabolism and insulin signaling, which may induce insulin resistance, lipid accumulation, and elevate diabetes risk [10,11,12]. In the immune system, DEHP/MEHP activates nuclear factor-kappa B (NF-κB) and mitogen-activated protein kinase (MAPK) pathways, triggering abnormal secretion of pro-inflammatory cytokines (e.g., IL-6 and TNF-α) alongside oxidative stress, resulting in immune imbalance and chronic inflammation [13,14].

In addition to these systems, recent studies indicate that DEHP exposure significantly impacts the skeletal system, potentially by disrupting the dynamic balance between osteoblasts and osteoclasts [15,16], inducing bone resorption via inflammatory factors [17], and promoting excessive reactive oxygen species (ROS) generation [15]. These mechanisms may increase the risk of environment-related bone diseases such as osteoporosis and osteonecrosis of the femoral head [18].

The skeletal system relies on a functional balance between osteoblasts and osteoclasts to maintain bone mass and microstructure [19,20]. This balance can be disrupted by multiple factors, including abnormal hormone levels [21], inflammatory responses and oxidative stress [22], energy metabolism disorders [23], and exposure to environmental toxicants [18], leading to degenerative or destructive bone conditions such as osteoporosis and osteonecrosis. Osteoarthritis (OA), osteonecrosis of the femoral head (ONFH), and osteoporosis (OP) are three common chronic bone diseases with complex pathogenesis [24,25,26]. OA is characterized by articular cartilage degeneration, osteophyte formation, and synovial inflammation, closely associated with chondrocyte apoptosis [27], overactivation of matrix-degrading enzymes (such as matrix metalloproteinases, MMPs) [28], and local chronic inflammation [25]. ONFH is an aseptic necrotic bone disease caused by interrupted blood supply to the bone tissue. Its clinical features primarily include femoral head collapse, trabecular bone fracture, and hip joint dysfunction [29]. The pathogenesis involves microvascular injury, lipid deposition, osteocyte apoptosis, and imbalance in bone remodeling processes [30,31]. OP is a metabolic bone disease characterized by reduced bone density and deteriorated bone microstructure [32], primarily due to inadequate osteogenesis and/or enhanced osteoclast activity, increasing bone fragility and fracture risk [33].

Existing research suggests DEHP affects bone homeostasis through various pathways, including NF-κB activation, ROS promotion, interference with hormone regulation (e.g., estrogen and parathyroid hormone), inhibition of osteoblast differentiation, and promotion of osteoclast activation [15,34,35]. These mechanisms are highly consistent with the cartilage inflammation in OA, osteocyte necrosis in ONFH, and bone loss in OP. However, related mechanisms still lack systematic integration and network interpretation at the target level.

**Figure 1:**
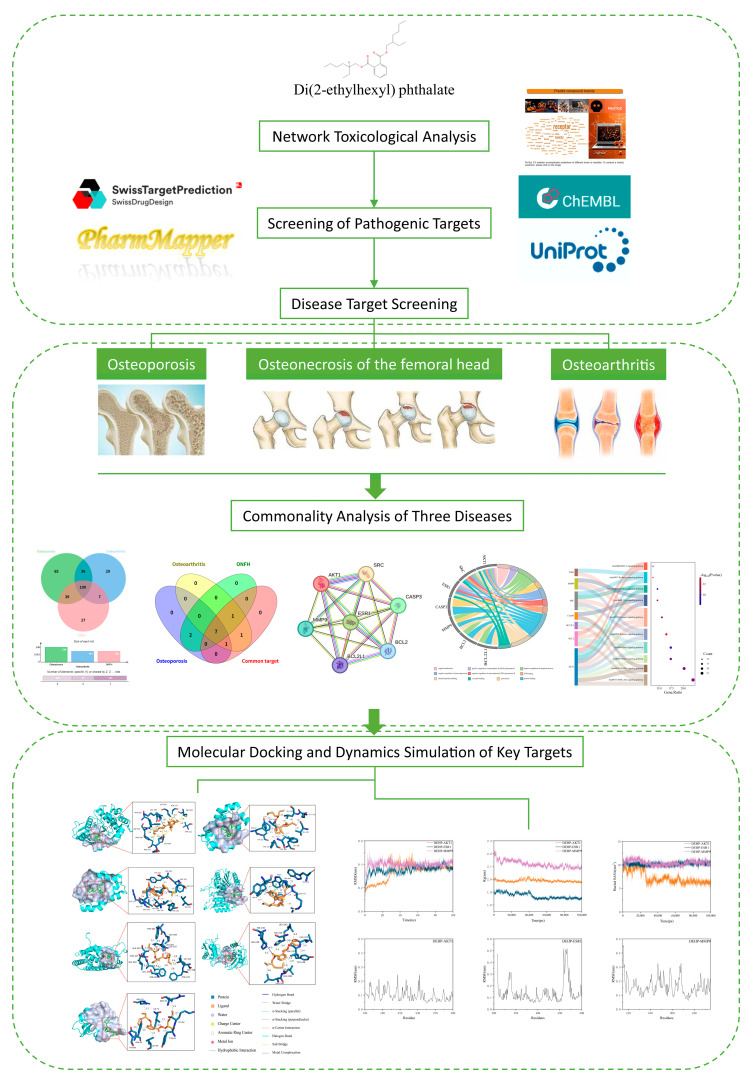
Analysis of the Workflow for Toxicological Mechanisms of DEHP in Bone Diseases.

Environmental network toxicology, an emerging branch of toxicology, integrates core concepts from toxicogenomics, systems biology, and network pharmacology. It emphasizes the multi-level integration of “chemical–target–pathway–disease” to predict and analyze the multi-organ and multi-pathway toxicity networks of environmental chemicals [36,37]. Building on the systematic research paradigm of “multi-component–multi-target–multi-pathway” proposed by Shah et al. and recognizing the critical role of molecular docking in validating network predictions [38], this study, based on the framework of network toxicology, aims to systematically construct a potential osteotoxicity network of DEHP (Figure 1). It focuses on its key targets and molecular pathways in three representative bone diseases—OA, ONFH, and OP—to reveal common mechanisms and differential characteristics of its effects, where the cross-disease analysis represents the main innovation of this work. This will provide new theoretical support and intervention clues for research on environmental pathogenic mechanisms of bone system diseases.

## 2. Results

### 2.1. Preliminary Toxicological Network Evaluation of DEHP

**Figure 2:**
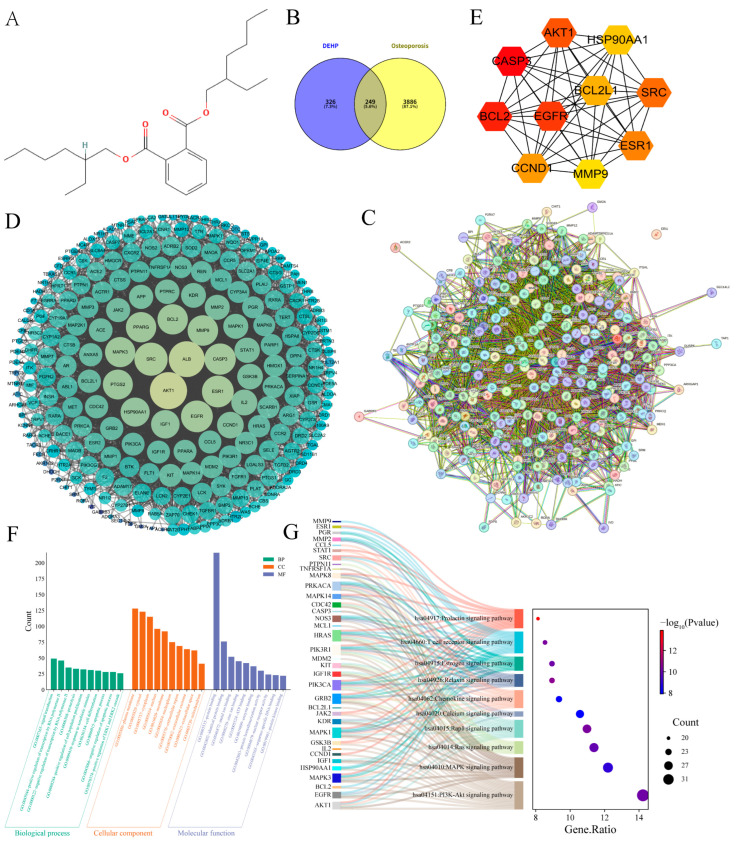
Screening and analysis of intersecting targets of DEHP and OP. (**A**) Chemical structure of the DEHP compound; (**B**) Venn diagram illustrating the overlapping targets between DEHP and OP; (**C**) PPI network of the 249 intersecting potential targets constructed using the STRING database; (**D**) PPI network of the intersecting targets constructed using Cytoscape 3.10.0 (the lighter the color and the larger the shape, the higher the degree value); (**E**) top 10 hub targets identified by the CytoHubba plugin(the darkness of the color corresponds to the magnitude of the degree value); (**F**) GO analysis results of the potential targets; (**G**) Sankey diagram displaying the key KEGG pathways and related genes of DEHP targets associated with OP.

To evaluate the toxicity of DEHP, its chemical data were obtained, including Chemical ID (CID: 8343), chemical structure, IUPAC Name (bis(2-ethylhexyl) benzene-1,2-dicarboxylate; methylsulfinylmethane), and SMILES (CCCCC(CC)COC(=O)C1=CC=CC=C1C(=O)OCC(CC)CCCC) (Figure 2A). A systematic analysis of DEHP’s toxicity was conducted using two major toxicity prediction platforms, ADMETlab 3.0 and ProTox-3.0. The results indicated that DEHP was predicted by the ProTox-3.0 database to exhibit inactive immunotoxicity and cytotoxicity, yet it possesses certain nephrotoxicity (probability 0.57) and significant carcinogenicity (probability 0.86), along with the ability to cross the blood–brain barrier (probability 0.87), suggesting its potential to interfere with central mechanisms regulating bone metabolism (Table S1). Simultaneously, ADMETlab 3.0 prediction results showed that DEHP has high skin sensitization potential (0.938) and eye irritation potential (0.979) and presents a moderate hERG inhibition risk (0.958), indicating its potential impact on cardiovascular–bone coupling processes. Furthermore, DEHP was predicted to strongly inhibit CYP enzymes (such as CYP3A4, CYP2C19, CYP2C8, etc.), which may affect the in vivo transport and metabolic stability of bone metabolism drugs (Table S2).

### 2.2. Impact of DEHP on Osteoporosis

A total of 575 DEHP-related targets were identified from the ChEMBL, SwissTargetPrediction, Similarity Ensemble Approach (SEA), and PharmMapper databases. From GeneCards and OMIM, 7355 and 629 OP-related targets were retrieved, respectively. After selecting targets above the threshold in the GeneCards database and removing duplicates, 4135 unique OP targets were obtained. Using Venny 2.1.0, 249 overlapping targets were identified (Figure 2B), representing the potential mechanisms by which DEHP induces OP. By importing these 249 intersecting targets into the STRING database to construct a PPI network, a network comprising 246 nodes and 4054 edges, with an average node degree of 33, was generated (Figure 2C). This highlights the complex interactions between DEHP-related toxicity targets and the pathophysiology of OP. To further investigate the toxic mechanisms of DEHP-induced OP, the TSV file exported from STRING was imported into Cytoscape 3.10.0 software for further analysis (Figure 2D). The MCC algorithm in CytoHubba was applied to identify and visualize the top 10 core genes: CASP3, BCL2, EGFR, AKT1, SRC, ESR1, CCND1, BCL2L1, HSP90AA1, and MMP9 (Figure 2E).

To elucidate the molecular mechanisms of DEHP-induced osteoporosis, we performed GO and KEGG enrichment analyses on 249 candidate targets. GO analysis revealed (Figure 2F) that DEHP significantly perturbs core biological processes directly relevant to bone homeostasis, including the regulation of cell proliferation (32 genes, *p* = 3.97 × 10^−13^), differentiation (30 genes, *p* = 2.55 × 10^−8^), apoptosis (28 genes, *p* = 6.29 × 10^−8^), and response to xenobiotic stimulus (31 genes, *p* = 1.13 × 10^−20^). The corresponding proteins are primarily localized to the plasma membrane, extracellular region, and nucleus, with molecular functions enriched in protein binding and kinase activity. This collectively suggests that DEHP may disrupt membrane receptor-mediated signal transduction and nuclear transcriptional regulation, thereby dysregulating bone cell fate decisions. KEGG pathway analysis further uncovered central signaling axes potentially targeted by DEHP. Significant enrichment was observed in pivotal pathways such as PI3K-Akt (35 genes, *p* = 7.84 × 10^−11^), MAPK (30 genes, *p* = 1.14 × 10^−9^), and calcium signaling (26 genes, *p* = 1.22 × 10^−8^). These pathways converge on key genes like AKT1, MAPK1/3, and EGFR, which coordinately regulate osteoblast survival, differentiation, and mineralization. The enrichment of the estrogen signaling pathway (22 genes, *p* = 7.66 × 10^−11^) indicates that DEHP might mimic or interfere with the bone-protective actions of endogenous hormones (Figure 2G). Furthermore, enrichment in immune-related pathways such as the T cell receptor signaling pathway (21 genes, *p* = 4.82 × 10^−11^) implies that DEHP could indirectly promote osteoclastogenesis by activating inflammatory-immune responses. In summary, we propose that DEHP collaboratively disrupts bone homeostasis through multiple interconnected mechanisms: by impairing PI3K-Akt/MAPK-dependent survival signaling, disturbing calcium homeostasis and estrogen-mediated metabolic balance, and potentially activating immune-bone crosstalk. These concerted actions shift the osteoblast–osteoclast equilibrium toward bone loss, ultimately promoting osteoporosis.

### 2.3. Impact of DEHP on Osteoarthritis

**Figure 3:**
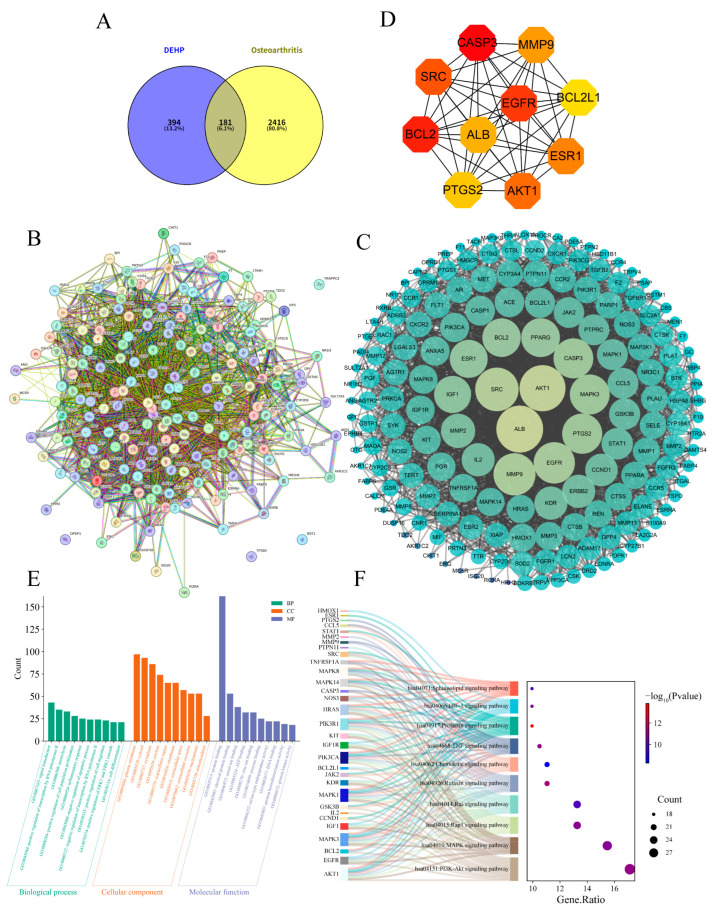
Screening and analysis of intersecting targets of DEHP and OA. (**A**) Venn diagram illustrating the overlapping targets between DEHP and OA; (**B**) PPI network of the 181 intersecting potential targets constructed using the STRING database; (**C**) PPI network of the intersecting targets constructed using Cytoscape 3.10.0 (the lighter the color and the larger the shape, the higher the degree value); (**D**) top 10 hub targets identified by the CytoHubba plugin; (**E**) GO analysis results of the potential targets(the darkness of the color corresponds to the magnitude of the degree value); (**F**) Sankey diagram displaying the key KEGG pathways and related genes of DEHP targets associated with OA.

After screening and integration from the GeneCards and OMIM databases, a total of 2597 OA-related targets were retrieved. Using Venny 2.1.0, 181 overlapping targets were identified (Figure 3A), representing the potential mechanisms by which DEHP induces OA. These targets were imported into the STRING database to construct a PPI network (Figure 3B), resulting in a network comprising 179 nodes and 2674 edges, with an average node degree of 29.9. This highlights the complex interactions between DEHP-related toxicity targets and OA pathophysiology. The TSV file exported from STRING was imported into Cytoscape 3.10.0 software for further analysis (Figure 3C). The top 10 core targets were selected based on the MCC algorithm (Figure 3D): CASP3, BCL2, EGFR, SRC, AKT1, ESR1, MMP9, ALB, PTGS2, and BCL2L1.

Gene Ontology (GO) enrichment analysis revealed (Figure 3E) strong mechanistic links to core cellular functions. The most significant enrichment was observed in signal transduction (GO:0007165, *p* = 5.67 × 10^−13^), driven by genes such as EGFR, AKT1, MAPK1, and STAT1, indicating their central role in propagating intracellular signals. Key biological processes, including positive regulation of transcription by RNA polymerase II (GO:0045944, *p* = 3.95 × 10^−9^), proteolysis (GO:0006508, *p* = 1.40 × 10^−15^), and inflammatory response (GO:0006954, *p* = 1.70 × 10^−12^), further support involvement in transcriptional control, protein degradation, and immune activation. Cellular component terms such as plasma membrane (GO:0005886, *p* = 4.70 × 10^−14^) and extracellular space (GO:0005615, *p* = 2.43 × 10^−17^) suggest these genes functionally support intercellular communication, while molecular function enrichments like protein binding (GO:0005515, *p* = 2.15 × 10^−9^) imply the assembly of multi-protein complexes essential for signaling. KEGG pathway analysis further delineated a coordinated network of signaling mechanisms. Significant enrichment was identified in the PI3K-Akt, MAPK, and JAK-STAT pathways, highlighting conserved regulatory axes governing cell survival and proliferation (Figure 3F). Co-enrichment in TNF, NF-κB, and HIF-1 signaling pathways points to integrated control of inflammatory and stress responses. Additional pathways, such as cytokine–cytokine receptor interaction and Chemokine signaling, further reflect specialized roles in immune modulation. Enrichment in Focal adhesion and ECM–receptor interaction also suggests alterations in cellular adhesion and microenvironment crosstalk. Collectively, these results position the identified genes within a functionally cohesive network that regulates proliferation, apoptosis, and inflammatory signaling through both canonical and context-specific mechanisms.

### 2.4. Impact of DEHP on Osteonecrosis of the Femoral Head

**Figure 4:**
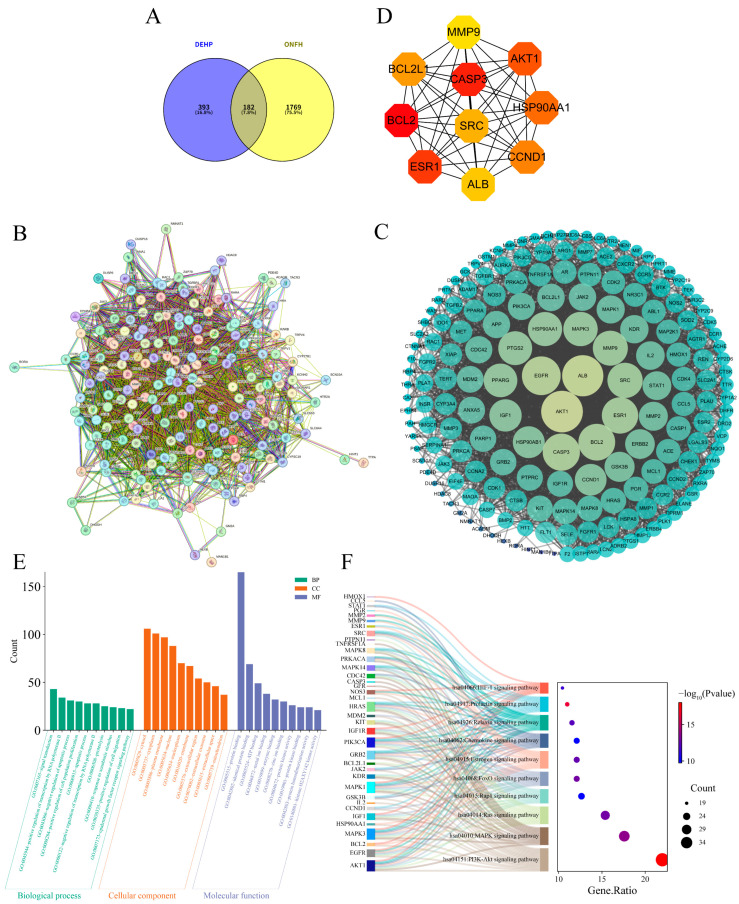
Screening and analysis of intersecting targets of DEHP and ONFH. (**A**) Venn diagram illustrating the overlapping targets between DEHP and ONFH; (**B**) PPI network of the 182 intersecting potential targets constructed using the STRING database; (**C**) PPI network of the intersecting targets constructed using Cytoscape 3.10.0 (the lighter the color and the larger the shape, the higher the degree value); (**D**) top 10 hub targets identified by the CytoHubba plugin; (**E**) GO analysis results of the potential targets(the darkness of the color corresponds to the magnitude of the degree value); (**F**) Sankey diagram displaying the key KEGG pathways and related genes of DEHP targets associated with ONFH.

After screening and integration from the GeneCards and OMIM databases, a total of 1951 ONFH-related targets were retrieved. Using Venny 2.1.0, 182 overlapping targets were identified (Figure 4A). These targets represent the potential mechanisms by which DEHP induces ONFH. These targets were imported into the STRING database to construct a PPI network (Figure 4B), resulting in a network comprising 182 nodes and 3463 edges, with an average node degree of 38.1. The TSV file exported from STRING was imported into Cytoscape 3.10.0 software for further analysis (Figure 4C). The top 10 core targets, potentially playing key roles in DEHP-induced ONFH toxicity, were selected based on the MCC algorithm (Figure 4D): BCL2, CASP3, ESR1, AKT1, HSP90AA1, CCND1, BCL2L1, SRC, ALB, and MMP9.

Based on the GO and KEGG enrichment analyses, the identified targets are mechanistically implicated in key signaling and regulatory networks. In the GO analysis (Figure 4E), biological processes such as signal transduction (GO:0007165, *p* = 6.93 × 10^−13^), transcriptional regulation by RNA polymerase II (GO:0045944, *p* = 1.66 × 10^−8^), and apoptotic regulation (GO:0043066, *p* = 7.06 × 10^−16^) were significantly enriched. Key molecules, including SRC, EGFR, AKT1, JAK2, MAPK1, and NR3C1, likely function as central nodes within these processes. Cellular component terms highlighted cytosol (GO:0005829), plasma membrane (GO:0005886), and extracellular regions, suggesting both intracellular and intercellular signaling roles. Molecular function was dominated by protein binding (GO:0005515), consistent with the formation of multi-protein signaling complexes. KEGG pathway analysis further reinforced these findings (Figure 4F), showing significant enrichment in the PI3K-Akt, MAPK, NF-κB, JAK-STAT, and calcium signaling pathways. Shared components such as PIK3R1, EGFR, and SRC suggest points of crosstalk between these pathways, potentially coordinating cellular responses in proliferation, stress, and immune modulation. These integrated results support a model in which the candidate genes collectively regulate signal transduction, transcriptional programs, and cell fate decisions, providing a mechanistic basis for the observed phenotypic outcomes.

### 2.5. Synergistic Analysis of Core Targets Across Three Bone Diseases

**Figure 5:**
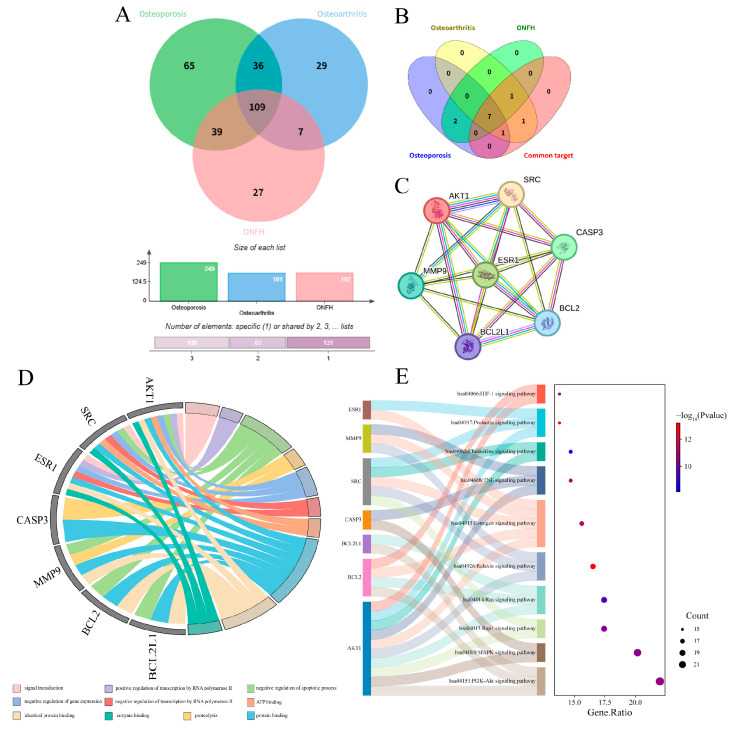
Comprehensive analysis of the core targets. (**A**) Venn diagram of the intersecting targets between DEHP and the three bone diseases (OP, OA, ONFH), accompanied by a statistical bar chart showing the number of targets for each disease; (**B**) Venn diagram illustrating the common core targets shared by the three bone diseases; (**C**) protein–protein interaction (PPI) network of the common core targets shared by the three diseases; (**D**) chord diagram of the Gene Ontology (GO) enrichment analysis for the common core targets; (**E**) Sankey diagram and bubble plot of the Kyoto Encyclopedia of Genes and Genomes (KEGG) pathway enrichment analysis for the common core targets shared by the three diseases.

Through comparative analysis of the core targets of OP, OA, and ONFH, a total of 109 common targets were identified (Figure 5A). These targets were imported into the STRING database to construct a PPI network. The TSV file exported from STRING was subsequently imported into Cytoscape 3.10.0 software for further analysis. The top 10 core targets, selected based on the MCC algorithm, were BCL2, BL2L1, MMP9, CASP3, PTGS2, EGFR, SRC, ALB, ESR1, and AKT1. Further cross-comparison with the respective core targets of the three individual diseases ultimately revealed 7 core targets common to all three diseases: AKT1, SRC, ESR1, CASP3, MMP9, BCL2, and BCL2L1 (Figure 5B,C).

Enrichment analysis results indicated that GO enrichment showed these common core targets are primarily involved in the regulation of apoptosis, inflammatory response, cell migration, angiogenesis, and bone remodeling-related processes. Cellular localization was concentrated in key compartments such as the plasma membrane, cytoplasm, nucleus, and exosomes. Molecular functions were significantly enriched in protein binding, kinase activity, and transcription factor binding (Figure 5D). KEGG pathway analysis revealed that the PI3K-Akt, MAPK, Ras, TNF, and estrogen signaling pathways were significantly enriched across all three disease types (Figure 5E), suggesting these signaling axes may constitute a common molecular network mediating DEHP-induced bone metabolism imbalance.

Comprehensively, DEHP likely promotes the occurrence and progression of chronic bone diseases such as OP, OA, and ONFH through a synergistic multi-target action. This involves simultaneously interfering with AKT1/SRC-mediated survival and proliferation signals, ESR1-related estrogen regulation, BCL2 family and CASP3-mediated apoptotic pathways, and MMP9-mediated matrix degradation.

### 2.6. Molecular Docking of DEHP with Core Targets Associated with Bone Diseases

**Figure 6:**
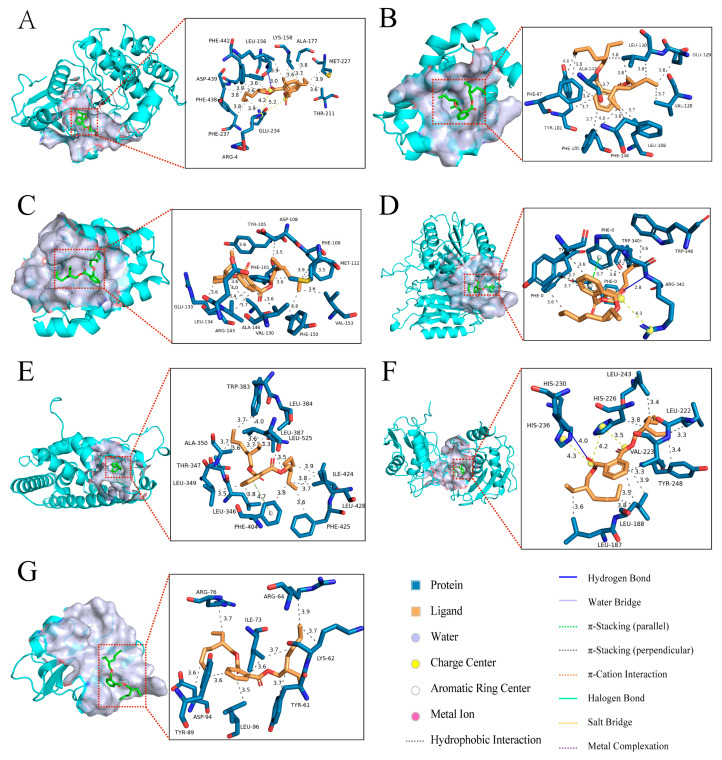
Molecular docking results showing the lowest binding energy conformations of DEHP with each core target. (**A**) DEHP and AKT1; (**B**) DEHP and BCL2L1; (**C**) DEHP and BCL2; (**D**) DEHP and CASP3; (**E**) DEHP and ESR1; (**F**) DEHP and MMP9; (**G**) DEHP and SRC.

Molecular docking was performed between DEHP and seven core targets related to bone diseases (Figure 6 and Table 1), including AKT1 (PDB ID: 3MVH), SRC (PDB ID: 1O43), ESR1 (PDB ID: 1SJ0), CASP3 (PDB ID: 1RHJ), MMP9 (PDB ID: 4H1Q), BCL2 (PDB ID: 4LXD), and BCL2L1 (PDB ID: 4EHR). The predicted binding affinities (kcal/mol) were −8.2, −5.0, −7.7, −6.5, −7.6, −6.8, and −6.3, respectively. A more negative binding affinity value indicates a stronger and more stable interaction between the ligand and the protein. The results demonstrate that AKT1 exhibited the strongest docking activity, showing the most favorable binding conformation among the multiple target genes analyzed. The docking simulations identified specific interactions, including hydrogen bonds and van der Waals forces, which are critical for understanding the binding mode and stability of DEHP with its core protein targets.

### 2.7. Molecular Dynamics Simulation Validation of Key Toxicity Targets

**Figure 7:**
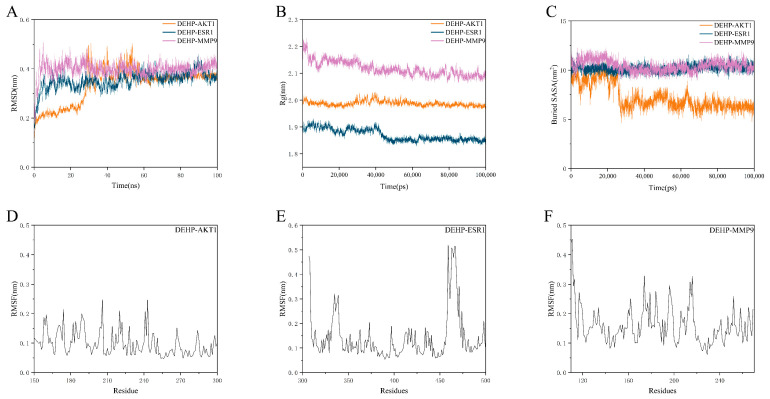
Molecular dynamics simulations of the protein-ligand complexes. (**A**) Root mean square deviation (RMSD) values of the protein-ligand complexes over simulation time; (**B**) radius of gyration (Rg) values of the protein-ligand complexes over simulation time; (**C**) solvent accessible surface area (SASA) values of the protein-ligand complexes over simulation time; (**D**–**F**) root mean square fluctuation (RMSF) values of the backbone atoms in the amino acid residues of the protein-ligand complexes over simulation time.

To further confirm the binding affinity of DEHP with the core targets, the three targets with the highest molecular docking binding energies were selected for MD simulations. The Root Mean Square Deviation (RMSD) was measured from the outset to assess protein-ligand conformational stability and atomic positional deviations relative to a reference structure (typically the initial or crystal structure). Generally, lower RMSD values indicate higher similarity to the reference structure and better conformational stability. Here, RMSD evaluated the equilibration of the simulated systems. As shown in Figure 7A, the DEHP-ESR1 and DEHP-MMP9 complexes reached equilibrium after 5 ns, fluctuating around 0.3 nm and 0.4 nm, respectively, while the DEHP-AKT1 complex reached equilibrium after 30 ns, fluctuating around 0.4 nm. Consequently, DEHP exhibited high stability when bound to AKT1, ESR1, and MMP9.

The Radius of Gyration (Rg) was analyzed to assess the overall compactness and folding stability of the protein. A structure is considered to have stable folding if its Rg remains consistent throughout the MD simulation. The Solvent Accessible Surface Area (SASA), which measures the proportion of the protein surface exposed to water, helps predict conformational changes during interactions. Further analysis revealed stable fluctuations in the Rg values for the DEHP-AKT1, DEHP-ESR1, and DEHP-MMP9 complexes throughout the simulation period. This indicates that DEHP did not induce significant expansion or contraction in AKT1, ESR1, or MMP9 (Figure 7B,C).

The Root Mean Square Fluctuation (RMSF) reflects the flexibility of amino acid residues in the protein. As shown in Figure 7D–F, the DEHP-AKT1, DEHP-ESR1, and DEHP-MMP9 complexes exhibited relatively low RMSF values (mostly below 3 Å), indicating lower flexibility and higher stability.

In summary, the DEHP-AKT1, DEHP-ESR1, and DEHP-MMP9 complexes demonstrated stable binding and favorable hydrogen bond interactions. This suggests that DEHP interacts effectively with the target proteins AKT1, ESR1, and MMP9.

## 3. Discussion

OP, OA, and ONFH are three common chronic bone diseases with complex pathogenesis, all closely associated with the disruption of bone metabolic homeostasis [39]. The health of bone tissue relies on a precise balance between osteoblasts and osteoclasts in processes such as proliferation, differentiation, and apoptosis, which is regulated by various factors, including inflammatory cytokines, hormonal signals, and extracellular matrix remodeling [40]. In recent years, environmental EDCs have garnered increasing attention as potential risk factors for bone damage. Among them, DEHP, due to its widespread environmental exposure and persistent bioaccumulation, has been demonstrated to affect bone metabolism by inducing inflammatory responses, interfering with estrogen signaling, and promoting apoptosis, among other pathways [15,17,41]. However, most current studies focus solely on the mechanism of DEHP’s action on a single bone disease, lacking a systematic analysis of its commonalities and differences across various bone disorders.

Based on the framework of environmental network toxicology, this study, for the first time, systematically integrated and compared the potential targets of DEHP across OP, OA, and ONFH. Through synergistic analysis of core targets, it revealed the common molecular network of DEHP in the pathogenesis of these three diseases. We identified 109 targets common to all three diseases from the PPI network and further pinpointed seven core intersection targets (AKT1, SRC, ESR1, CASP3, MMP9, BCL2, and BCL2L1). These targets not only exhibited high network centrality in the individual analysis of each disease but also collectively participated in key pathological processes such as the regulation of apoptosis, amplification of inflammatory signals, cell migration, and matrix degradation. KEGG enrichment results indicated that the PI3K-Akt, MAPK, Ras, TNF, and estrogen signaling pathways were significantly enriched in all three diseases, suggesting that DEHP may induce a cross-disease pattern of bone metabolism imbalance through synergistic interference via multiple pathways and targets. Although pathways such as PI3K-Akt, MAPK, and TNF are frequently enriched, several key genes within them play essential roles in bone metabolism and DEHP-induced toxicity. DEHP may compromise osteogenic function and promote bone resorption in OP by suppressing AKT1, a core gene regulating osteoblast survival and differentiation. At the same time, it enhances osteoclast activity and accelerates bone loss by activating SRC, a gene involved in osteoclastogenesis. In the MAPK pathway, DEHP promotes inflammatory responses and matrix degradation through MAPK1/3, with MMP9 playing a critical role in cartilage breakdown in OA. Within the TNF pathway, DEHP activates NF-κB, thereby exacerbating osteoclast formation and bone resorption—a mechanism strongly linked to ONFH. Furthermore, DEHP disrupts the apoptosis-related genes BCL2 and CASP3, promoting osteocyte death and contributing to the development of osteonecrosis. In summary, DEHP disrupts bone homeostasis by synergistically interfering with multiple gene functions, amplifying inflammatory responses, and facilitating matrix degradation, collectively driving the progression of skeletal disorders. This finding indicates that DEHP’s effect is not a single event but rather causes systemic damage through a “multi-target coupling network,” which contrasts sharply with the traditional toxicological “single-target–single-effect” model and better reflects the real-world scenario of complex pathology under environmental exposure.

The results of the synergistic analysis not only reveal the common mechanistic basis of DEHP’s action across different bone diseases but also provide potential molecular targets for multi-disease intervention. For instance, AKT1 and SRC play central roles in cell survival and signal transduction, and their dysregulation may lead to increased osteoblast apoptosis and inhibited bone formation [42,43]. ESR1-mediated estrogen signaling is closely related to the maintenance of bone density, and the weak estrogen-like effect of DEHP may weaken this protective mechanism [44,45,46]. Abnormal regulation of the BCL2 family can directly drive osteocyte programmed death [47,48,49]. Once abnormally activated, CASP3 can directly cleave key substrates such as cytoskeletal proteins and PARP1, irreversibly initiating the programmed death cascade [50]. In bone cells, deficiency or inhibition of CASP3 significantly reduces the proliferation and osteogenic differentiation of bone marrow stromal stem cells (BMSSCs), leading to decreased bone mass [51,52]. Conversely, microenvironmental stimuli like oxidative stress and TNF-α activate CASP3 through mitochondrial or death receptor pathways, rapidly inducing apoptosis in chondrocytes, osteoblasts, and osteocytes, thereby driving bone-destructive processes such as OP or arthritis [53,54]. MMP9 promotes matrix degradation, accelerating the structural destruction of bone tissue [55,56]. The synergistic imbalance of these targets may constitute the common molecular pathological basis for various bone diseases following DEHP exposure. Furthermore, this study also revealed differential effects of DEHP across different bone diseases: it primarily disrupts the osteoblast/osteoclast balance in OP, more prominently promotes inflammation and cartilage matrix degradation in OA, and is closely related to angiogenesis and ischemic injury in ONFH. This model of “commonality and differential characteristics” provides a novel perspective for gaining deeper insights into the relationship between environmental toxicants and complex bone diseases.

This study introduces several conceptual and methodological innovations compared to existing research on DEHP. A key advance lies in the cross-disease comparison of DEHP-induced bone disorders—namely OP, OA and ONFH. While earlier investigations have largely examined DEHP toxicity within the context of a single bone disease, our work systematically delineates both common and distinct pathological mechanisms across these conditions. In contrast to prior studies that often focused on isolated pathways, our multi-pathway analysis more accurately captures the complexity of DEHP’s impact on bone metabolism and highlights its interconnected toxicity network across different bone diseases. This cross-disease synergistic perspective constitutes the core novelty of our study, providing new insights into how environmental endocrine disruptors such as DEHP may concurrently influence multiple bone disorders. From a public health perspective, this study holds significant practical importance. As one of the most widely used plasticizers, DEHP is ubiquitous in food packaging, medical devices, and daily necessities, leading to long-term exposure in the population. Compared to the reproductive and metabolic system damage primarily focused on in existing studies, this study emphasizes the skeletal system as an emerging target of DEHP, suggesting its potential threat to bone health. Future research in environmental health and bone disease epidemiology should adopt a holistic framework integrating the “bone–metabolism–immunity–endocrinology” systems to systematically assess the risks of environmental pollutants to bone health.

Furthermore, from a translational research standpoint, the seven common core targets identified in this study not only serve as molecular evidence of DEHP’s osteotoxicity but could also become potential therapeutic targets for intervening in environmentally related bone diseases. For example, small-molecule inhibitors targeting the AKT1/SRC signaling axis, specific MMP9 inhibitors, or enhancing the protective estrogenic effect by modulating the ESR1 pathway could all be promising strategies to counteract DEHP-induced bone toxicity. Although this study provides important insights into the molecular mechanisms of DEHP-induced bone toxicity, several limitations should be noted. First, the findings are based on computational models and predictive analyses, and their accuracy needs to be verified by subsequent experiments. Second, due to the highly dynamic and complex regulatory networks in biological systems, relying solely on network toxicology and molecular docking methods may not fully simulate real biological interactions, which could affect the depth of biological interpretation of the results. Simultaneously, combining the development of green alternative plasticizers [57] and drug repurposing could help translate basic research findings into practical prevention and control measures, providing new solutions for public health.

In summary, this study not only confirms the multi-target disruptive effects of DEHP on OP, OA, and ONFH but also constructs a core interaction network across these diseases at the molecular level through synergistic analysis. The methodological advantage of this approach provides a new perspective for environmental toxicology research. By identifying key nodes and signaling pathways common to multiple diseases, a more comprehensive assessment of the systemic risks posed by environmental chemicals to bone health can be achieved, providing a theoretical basis for future combined prevention and intervention strategies targeting multiple bone diseases.

## 4. Materials and Methods

### 4.1. Network Toxicological Analysis of DEHP

The SMILES sequence of DEHP was retrieved from the PubChem database (https://pubchem.ncbi.nlm.nih.gov/ (accessed on 20 June 2025)). This sequence was subsequently input into two databases, ADMETlab 3.0 (https://admetlab3.scbdd.com/server/evaluationCal (accessed on 20 June 2025)) [58] and ProTox 3.0 (https://tox.charite.de/ (accessed on 20 June 2025)) [59], to obtain toxicity predictions and analyses.

### 4.2. Collection of DEHP Targets

DEHP was searched, identified, and verified in the PubChem database to obtain its CID, structure, molecular formula, molecular weight, IUPAC name, and SMILES number. The SMILES notation was uploaded to SwissTargetPrediction (http://www.swisstargetprediction.ch/ (accessed on 23 June 2025)) [60] and the Similarity Ensemble Approach (SEA) (https://sea.bkslab.org/ (accessed on 23 June 2025)) [61], selecting Homo sapiens and downloading all predicted targets with a probability greater than 0. Simultaneously, target information was retrieved from the ChEMBL database (https://www.ebi.ac.uk/chembl/ (accessed on 23 June 2025)) [62] and PharmMapper (https://www.lilab-ecust.cn/pharmmapper/ (accessed on 24 June 2025)) [63], with the “Select Targets Set” option set to “Human Protein Targets Only (v2010, 2241)”. Results from all four databases were integrated, deduplicated, and the target names were standardized using the UniProt database (https://www.uniprot.org/ (accessed on 24 June 2025)) [64]. After combining and deduplicating the prediction results, the final target library for DEHP was established.

### 4.3. Construction of Disease Target Library

The target library for chronic bone system diseases was primarily constructed using the GeneCards (https://www.genecards.org/ (accessed on 25 June 2025)) [65] and OMIM (https://www.omim.org/ (accessed on 25 June 2025)) databases. These databases were searched using the keywords “Osteoporosis”, “necrosis of femoral head”, and “osteoarthritis” to retrieve genes associated with human diseases. To ensure the obtained genes were highly relevant to chronic bone system diseases and DEHP effects, the “score” threshold in GeneCards was set to the median value, and genes with a “score” above the median were selected. Finally, the target names were standardized using the UniProt database, after which targets from both databases were merged and deduplicated.

### 4.4. Intersection of DEHP Targets and Disease Targets

The intersection between compound targets and disease targets was identified using the Venn diagram tool (https://bioinfogp.cnb.csic.es/tools/venny/ (accessed on 15 July 2025)). The DEHP target library and the bone system disease target library were uploaded to the website, and the results were obtained after submission.

### 4.5. Construction of Protein–Protein Interaction (PPI) Network

The STRING database (https://cn.string-db.org/ (accessed on 23 July 2025)) [66] was used to construct the PPI network. The overlapping targets from the Venn diagram were imported into the database, with the species set to “Homo sapiens” and a minimum required interaction score of ≥0.4, to generate the PPI network map. The results from STRING were imported into Cytoscape 3.10.0 (Cytoscape Consortium, San Francisco, California, USA) [67] software for network visualization and analysis. Topological properties were calculated to generate the PPI network. The MCODE 1.6.1 (Gary Bader (University of Toronto), Toronto, Ontario, Canada) [68] plugin was first used to identify the most significant clusters of interacting nodes. Subsequently, the top 10 significant genes were screened using the MCC algorithm from the CytoHubba plugin. Finally, the intersection between the targets contained within the significant modules identified by MCODE and the targets predicted by CytoHubba 0.1 (Institute of Information Science, Academia Sinica, Taiwan, Taiwan, China) [69] was taken to determine the hub genes.

### 4.6. GO and KEGG Pathway Enrichment Analysis

The DAVID database (https://davidbioinformatics.nih.gov/ (accessed on 23 July 2025)) [70] was used for Gene Ontology (GO) term (Biological Process, Molecular Function, and Cellular Component) and Kyoto Encyclopedia of Genes and Genomes (KEGG) pathway enrichment analyses to investigate the pathways involved in chronic bone system diseases. This analysis aimed to elucidate and highlight the important signaling pathways involved in the biological processes. Visual analysis was conducted to effectively interpret and present the results of the GO and KEGG analyses, with SRplot (https://www.bioinformatics.com.cn/, (accessed on 25 July 2025)) used for generating reference graphs.

### 4.7. Molecular Docking

Molecular docking technology was employed to analyze the intermolecular interactions between DEHP and core target proteins. In this study, DEHP was used as the ligand for molecular docking. The top 7 hub targets with the highest degree values and overlap from the PPI network were selected as receptors. The 3D structure of the small molecule ligand was obtained from PubChem, and the 3D structures of the core proteins were predicted using AlphaFold 3 (https://alphafoldserver.com/ (accessed on 1 August 2025)) [71]. PyMOL 2.3.4 (Schrödinger Inc., New York, USA) was used to remove water molecules and original ligands from the target proteins. The prepared proteins were then imported into AutoDockTools 1.5.6 (Scripps Research Institute, La Jolla, California, USA) for hydrogenation, charge calculation, and non-polar hydrogen combination. After defining the grid box size and genetic algorithm parameters, AutoDock Vina was used to perform 50 molecular docking runs each to generate a broader range of ligand-target conformations. The results were visually analyzed using PyMOL and PLIP (https://plip-tool.biotec.tu-dresden.de/plip-web/plip/index (accessed on 1 August 2025)) [72]. The docking simulations identified specific interactions, including hydrogen bonds and hydrophobic interactions, which are crucial for understanding the binding mode and stability of DEHP with its core protein targets.

### 4.8. Molecular Dynamics (MD) Simulation

Gromacs 2022 [73] was used to perform 100 ns MD simulations of the complexes. The AMBER14SB force field and TIP3P water model were applied to the proteins, while the GAFF force field was used for the small molecules. The files for the protein and small molecule ligand were merged to construct the simulation system for the complex. Simulations were conducted under constant temperature and pressure with periodic boundary conditions. During the MD simulations, all bonds involving hydrogen were constrained using the LINCS algorithm, with an integration time step of 2 fs. Electrostatic interactions were calculated using the Particle-Mesh Ewald (PME) method with a cutoff set to 1.2 nm. The non-bonded interaction cutoff was set to 10 Å, updated every 10 steps. The V-rescale temperature coupling method was used to maintain the simulation temperature at 298 K, and the Berendsen method was used to maintain the pressure at 1 bar. After 100 ps of NVT and NPT equilibrium simulations at 298 K, a 100 ns MD simulation was performed on the complex system, saving conformations every 10 ps. After the simulation, VMD and PyMOL were used to analyze the simulation trajectories.

## 5. Conclusions

By integrating environmental network toxicology with molecular docking, this study systematically investigated the potential toxic mechanisms of DEHP in three bone-related diseases: OP, OA, and ONFH. These findings provide novel insights into the role of environmental EDCs in the pathogenesis of chronic bone diseases, thereby laying a theoretical foundation for the subsequent development of drugs targeting the clearance of EDCs or the blockade of their toxic effects. Furthermore, intervention strategies based on key targets and research on combined environmental exposures are expected to open new avenues for the prevention and treatment of environmentally associated bone diseases.

## Figures and Tables

**Table 1:** Molecular docking analysis of binding affinity between DEHP and core targets.

Target Name	PDB ID	Binding Energy (kcal/mol)	Three-Dimensional Coordinates	Key Interacting Residues	RMSD
AKT1	3MVH	−8.2	center_x = 18.326 center_y = −2.446 center_z = 28.06	LYS158, ALA177, MET227, THR211, GLU234, PHE237, PHE438, ASP439, PHE442, LEU156	2.479
BCL2L1	4EHR	−6.3	center_x = 13.921 center_y = 30.454 center_z = 7.391	LEU130, GLU129, VAL126, LEU108, PHE146, PHE105, TYR101, PHE97, ALA142,	3.301
BCL2	4LXD	−6.8	center_x = 23.766 center_y = 32.657 center_z = 10.164	TYR105, ASP108, PHE109, MET112, VAL153, PHE150, VAL130, ALA146, ARG143, LEU134, GLU133, PHE101	4.390
CASP3	1RHJ	−6.5	center_x = −96.163 center_y = 25.222 center_z = 45.774	PHE0, TRP340, TRP348, ARG341, TYR338	4.004
ESR1	1SJ0	−7.7	center_x = 31.406 center_y = −1.491 center_z = 25.297	TRP383, LEU384, LEU387, LEU525, ILE424, LEU428, PHE425, PHE404, LEU346, LEU349, THR347, ALA350	2.494
MMP9	4H1Q	−7.6	center_x = 29.306 center_y = −5.638 center_z = 25.733	LEU243, LEU222, TYR248, LEU188, LEU187, HIS236, HIS230, HIS226, VAL223	5.571
SRC	1O43	−5.0	center_x = 19.624 center_y = 22.011 center_z = 21.789	ARG76, ARG64, ILE73, LYS62, TYR61, LEU96, ASP94, TYR89	1.509

## Data Availability

The additional data supporting the manuscript are available from the corresponding author upon request.

## Supplementary Materials

The following supporting information can be downloaded at: https://www.mdpi.com/article/10.3390/ijms262210895/s1.

## Abbreviations

The following abbreviations are used in this manuscript:

DEHP	Di(2-ethylhexyl) phthalate
OP	Osteoporosis
OA	Osteoarthritis
ONFH	Osteonecrosis of the femoral head
PPI	Protein–protein interaction
GO	Gene Ontology
KEGG	Kyoto Encyclopedia of Genes and Genomes
MD	Molecular Dynamics
PVC	Polyvinyl Chloride
EDC	Endocrine-Disrupting Chemical
MEHP	Mono(2-ethylhexyl) phthalate
AR	Androgen receptor
ER	Estrogen receptor
PPARα/γ	Peroxisome proliferator-activated receptors
NF-κB	Nuclear factor-kappa B
MAPK	Mitogen-activated protein kinase
ROS	reactive oxygen species
SEA	Similarity Ensemble Approach
IUPAC	International Union of Pure and Applied Chemistry
MCC	Maximal Clique Centrality
RMSD	Root Mean Square Deviation
Rg	Radius of Gyration
SASA	Solvent Accessible Surface Area
RMSF	Root Mean Square Fluctuation
PME	Particle-Mesh Ewald
